# Supplementary material for: Current Research Status and Implication for Further Study of Real-World Data on East Asian Traditional Medicine for Heart Failure: A Scoping Review
Source: Healthcare (Basel). 2023 Dec 27;12(1):61. doi: 10.3390/healthcare12010061 (PMC10779411; doi:10.3390/healthcare12010061)
Supplement: Supplementary file 1 [file healthcare-12-00061-s001.zip › healthcare-2683139-supplementary.pdf]

Supplementary Table S1. Literature searching strategy

	**Medline via PubMed (20220510) searches**	**Results**
#1	(“heart failure” [Mesh] OR “heart failure” [tiab] OR “cardiac failure” [tiab] OR “myocardial failure” [tiab] OR “heart insufficiency" [tiab] OR “cardiac insufficiency” [tiab] OR “Cardiomyopathies” [Mesh] OR “Cardiomyopathies” [tiab] OR “Ventricular Dysfunction” [Mesh] OR “Ventricular Dysfunction” [tiab] OR “Heart Decompensation” [tiab] OR “Decompensation, Heart” [tiab] OR “Congestive Heart Failure” [tiab] OR “Heart Failure, Congestive” [tiab])	362,447
#2	(“Medicine, Chinese Traditional” [MH] OR “Medicine, Kampo” [MH] OR “Medicine, Korean Traditional” [MH] OR “traditional Korean medicine” [TIAB] OR “traditional Chinese medicine” [TIAB] OR “traditional oriental medicine" [TIAB] OR "Kampo medicine” [TIAB] OR “Chinese patent medicine"[TIAB] OR Acupuncture [MH] OR “Acupuncture Therapy” [MH] OR Auriculotherapy [MH] OR “Acupuncture, Ear” [MH] OR Acupressure [MH] OR Electroacupuncture [MH] OR “Acupuncture Points” [MH] OR “dry needling” [MH] OR acupuncture [TIAB] OR acupressure [TIAB] OR acupoint* [TIAB] OR “trigger point” [TIAB] OR “dry needling” [TIAB] OR “ear acupuncture” [TIAB] OR electroacupuncture [TIAB] OR electro-acupuncture [TIAB] OR “scalp acupuncture” [TIAB] OR “Head acupuncture” [TIAB] OR “acupotomy” [TIAB] OR “fire needling” [TIAB] OR “thread-embedding therapy” [TIAB] OR Moxibustion [MH] OR moxibustion [TIAB] OR moxa [TIAB] OR “warm needling” [TIAB] OR “Cupping Therapy” [MH] OR “cupping therapy” [TIAB] OR “Complementary Therapies” [MH] OR “complementary medicine" [TIAB] OR “alternative medicine” [TIAB] OR “herbal medicine” [TIAB] OR “herbal decoction” [TIAB] OR "herbal prescription” [TIAB] OR “Pharmacoacupuncture” [TIAB] OR Pharmaco-acupuncture [TIAB] OR Pharmacopuncture [TIAB])	276,584
#3	(“Epidemiologic Studies” [Mesh] OR “Retrospective cohort” [TIAB] OR “Cohort studies” [Mesh] OR “cohort studies” [TIAB] OR “Cohort Study” [TIAB] OR “Studies, Cohort” [TIAB] OR “Study, Cohort” [TIAB] OR ”Cohort Studies” [TIAB] OR “Case-Control studies” [Mesh] OR “Case-control studies” [TIAB] OR “Case-Control Study” [TIAB] OR “Cross-sectional studies” [Mesh] OR “Cross-sectional studies” [TIAB] OR “Cross-Sectional Study” [TIAB] OR “Insurance Claim Review” [Mesh] OR “Insurance Claim Review” [TIAB] OR “nationwide study” [TIAB] OR “nationwide cohort” [TIAB] OR “Sample cohort”[TIAB] OR “claim data” [TIAB] OR “national health insurance” [TIAB] OR “national health information” [TIAB] OR “population based” [TIAB] OR “matched cohort” [TIAB] OR “Propensity Score” [Mesh] OR “Propensity score” [TIAB] OR “health insurance review” [TIAB] OR “claim database” [TIAB] OR “claims database” [TIAB] OR “real-world”[TIAB] OR “real world” [TIAB] OR “Hospital information system” [TIAB])	3,239,816
#4	(#1 AND #2 AND #3)	112
#5		

	**EMBASE via Elsevier (20220525) searches**	**Results**
#1	‘Heart failure’/exp OR ‘heart failure’: ab,ti OR ‘cardiac failure’/exp OR ‘cardiac failure’: ab,ti OR ‘myocardial failure’exp OR ‘myocardial failure’: ab,ti OR ‘heart insufficiency’/exp OR 'heart insufficiency’: ab,ti OR ‘cardiac insufficiency’/exp OR ‘cardiac insufficiency’: ab,ti OR Cardiomyopathies: ab,ti OR Cardiomyopathies/exp OR ‘Heart Decompensation’/exp OR ‘Heart Decompensation’: ab,ti OR ‘Decompensation, Heart’/exp OR ‘Decompensation, Heart’: ab,ti OR ‘Congestive Heart Failure’/exp OR ‘Congestive Heart Failure’: ab,ti OR ‘Heart Failure, Congestive’/exp OR ‘Heart Failure, Congestive’: ab,ti	751,153
#2	‘Chinese medicine’/exp OR ‘chinese medicine’: ab,ti OR ‘kampo medicine’/exp OR ‘kampo medicine’: ab,ti OR ‘kampo medicine (drug)’/exp OR ‘kampo medicine (drug)’: ab,ti OR ‘korean medicine’/exp OR ‘korean medicine’: ab,ti OR ‘oriental medicine’/exp OR ‘oriental medicine’: ab,ti acupuncture/exp OR acupuncture: ab,ti OR ‘acupuncture therapy’: ab,ti OR	149,884

	'auricular acupuncture'/exp OR 'auricular acupuncture': ab,ti OR 'ear acupuncture': ab,ti OR auriculotherapy: ab,ti OR acupressure/exp OR acupressure: ab,ti OR electroacupuncture/exp OR electroacupuncture: ab,ti OR 'electro-acupuncture': ab,ti OR 'acupuncture point'/exp OR 'acupuncture point': ab,ti OR 'body meridian'/exp OR 'body meridian': ab,ti OR acupoint: ab,ti OR 'trigger point'/exp OR 'trigger point': ab,ti OR 'dry needling'/exp OR 'dry needling': ab,ti OR moxibustion/exp OR moxibustion: ab,ti OR moxa: ab,ti OR 'scalp acupuncture'/exp OR 'scalp acupuncture': ab,ti OR 'Head acupuncture'/exp OR 'Head acupuncture': ab,ti OR 'Acupotomy'/exp OR 'Acupotomy': ab,ti OR 'Fire needling'/exp OR 'fire needling': ab,ti OR 'thread-embedding therapy'/exp OR 'thread-embedding therapy': ab,ti OR 'warm needling': ab,ti OR 'cupping therapy'/exp OR 'cupping therapy': ab,ti OR 'complementary therapy'/exp OR 'complementary therapy': ab,ti OR 'complementary medicine': ab,ti OR 'alternative medicine'/exp OR 'alternative medicine': ab,ti OR 'herbal medicine'/exp OR 'herbal medicine': ab,ti OR 'herbal decoction': ab,ti OR 'Pharmacoacupuncture': ab,ti OR 'Pharmaco-acupuncture': ab,ti OR 'Pharmacopuncture': ab,ti	
#3	'Epidemiologic Studies': ab,ti OR 'Retrospective cohort': ab,ti OR 'Cohort analysis'/exp OR 'cohort analysis': ab,ti OR 'Cohort Study': ab,ti OR 'Studies, Cohort': ab,ti OR 'Study, Cohort': ab,ti OR 'Cohort Studies': ab,ti OR 'case control study'/exp OR 'case control study': ab,ti OR 'Case-Control Study': ab,ti OR 'cross-sectional study'/exp OR 'cross-sectional study': ab,ti OR 'Insurance Claim Review': ab,ti OR 'nationwide study': ab,ti OR 'nationwide cohort': ab,ti OR 'Sample cohort': ab,ti OR 'claim data': ab,ti OR 'national health insurance': ab,ti OR 'national health information': ab,ti OR 'population based': ab,ti OR 'matched cohort': ab,ti OR 'Propensity Score': ab,ti OR 'health insurance review': ab,ti OR 'claim database': ab,ti OR 'claims database': ab,ti OR 'real-world': ab,ti OR 'real world': ab,ti OR 'Hospital information system': ab,ti	1,890,065
#4	(#1 AND #2 AND #3)	71

	**Cochrane Library: Cochrane Reviews (20220525) searches**	**Results**
#1	MeSH descriptor: [Heart Failure] explode all trees OR (heart failure): ti,ab,kw OR (cardiac failure): ti,ab,kw OR (myocardial failure): ti,ab,kw OR (heart insufficiency): ti,ab,kw (cardiac insufficiency): ti,ab,kw OR (Cardiomyopathies): ti,ab,kw OR MeSH descriptor: [Cardiomyopathies] explode all trees OR MeSH descriptor: [Ventricular Dysfunction] explode all trees OR (Ventricular Dysfunction): ti,ab,kw OR (Heart Decompensation): ti,ab,kw OR (Decompensation, Heart): ti,ab,kw OR (Congestive Heart Failure): ti,ab,kw OR (Heart Failure, Congestive): ti,ab,kw	48094
#2	MeSH descriptor: [Medicine, Chinese Traditional] explode all trees OR MeSH descriptor: [Medicine, Kampo] explode all trees OR MeSH descriptor: [Medicine, Korean Traditional] explode all trees OR (traditional Korean medicine): ti,ab,kw OR (traditional Chinese medicine): ti,ab,kw OR (traditional oriental medicine): ti,ab,kw OR (Kampo medicine): ti,ab,kw OR (Chinese patent medicine): ti,ab,kw OR MeSH descriptor: [Acupuncture] explode all trees OR MeSH descriptor: [Acupuncture Therapy] explode all trees OR MeSH descriptor: [Auriculotherapy] explode all trees OR MeSH descriptor: [Acupuncture, Ear] explode all trees OR MeSH descriptor: [Acupressure] explode all trees OR MeSH descriptor: [Electroacupuncture] explode all trees OR MeSH descriptor: [Acupuncture Points] explode all trees OR MeSH descriptor: [Dry Needling] explode all trees OR (acupuncture): ti,ab,kw OR (acupressure): ti,ab,kw OR (acupoint): ti,ab,kw OR (trigger point): ti,ab,kw OR (dry needling): ti,ab,kw OR (ear acupuncture): ti,ab,kw OR (electroacupuncture): ti,ab,kw OR (electro-acupuncture): ti,ab,kw OR (scalp acupuncture):ti,ab,kw OR (Head acupuncture): ti,ab,kw OR (acupotomy): ti,ab,kw OR (fire needling): ti,ab,kw OR (thread-embedding therapy): ti,ab,kw OR MeSH descriptor: [Moxibustion] explode all trees OR (moxibustion):ti,ab,kw OR (moxa): ti,ab,kw OR (warm needling): ti,ab,kw OR MeSH descriptor: [Cupping Therapy] explode all trees OR (cupping therapy): ti,ab,kw OR MeSH descriptor: [Complementary Therapies] explode all trees OR (cupping therapy): ti,ab,kw OR MeSH descriptor: [Complementary Therapies] explode all trees OR (complementary medicine): ti,ab,kw OR (alternative medicine): ti,ab,kw OR (herbal medicine): ti,ab,kw OR	49844

	(herbal decoction): ti,ab,kw OR (herbal prescription): ti,ab,kw OR (Pharmacoacupuncture): ti,ab,kw OR (Pharmaco-acupuncture): ti,ab,kw OR (Pharmacopuncture): ti,ab,kw	
#3	MeSH descriptor: [Epidemiologic Studies] explode all trees OR (Retrospective cohort): ti,ab,kw OR MeSH descriptor: [Cohort Studies] explode all trees OR (cohort studies): ti,ab,kw OR (Cohort Study): ti,ab,kw OR (Studies, Cohort): ti,ab,kw OR (Study, Cohort): ti,ab,kw OR (Cohort Studies): ti,ab,kw OR MeSH descriptor: [Case-Control Studies] explode all trees OR (Case-control studies): ti,ab,kw OR (Case-Control Study): ti,ab,kw OR MeSH descriptor: [Cross-Sectional Studies] explode all trees OR (Cross-sectional studies): ti,ab,kw OR (Cross-Sectional Study): ti,ab,kw OR MeSH descriptor: [Insurance Claim Review] explode all trees OR (Insurance Claim Review): ti,ab,kw OR (nationwide study): ti,ab,kw OR (nationwide cohort): ti,ab,kw OR (Sample cohort): ti,ab,kw OR (claim data): ti,ab,kw OR (National Health Insurance): ti,ab,kw OR (national health information): ti,ab,kw OR (population based): ti,ab,kw OR (matched cohort): ti,ab,kw OR MeSH descriptor: [Propensity Score] explode all trees OR (Propensity score): ti,ab,kw OR (health insurance review): ti,ab,kw OR (claim database): ti,ab,kw OR (claims database): ti,ab,kw OR (real-world): ti,ab,kw OR (real world): ti,ab,kw OR (Hospital information system): ti,ab,kw	272043
#4	#1 and #2 and #3	68

	**CINAHL plus with full text (20220523) searches**	**Results**
#1	Heart failure OR cardiac failure OR myocardial failure OR heart insufficiency OR cardiac insufficiency OR Cardiomyopathies OR Heart Decompensation OR Congestive Heart Failure OR Ventricular Dysfunction	25,774
#2	Traditional Chinese medicine OR traditional oriental medicine OR Kampo medicine OR traditional Korean medicine OR Chinese patent medicine OR Acupuncture OR Acupuncture Therapy OR Auriculotherapy OR Acupressure OR Electroacupuncture OR Acupuncture Points OR dry needling OR acupoint* OR trigger point OR dry needling OR ear acupuncture OR electro-acupuncture OR scalp acupuncture OR Head acupuncture OR acupotomy OR fire needling OR thread-embedding therapy OR Moxibustion OR moxa OR warm needling OR Cupping Therapy OR Complementary Therapies OR complementary medicine OR alternative medicine OR herbal medicine OR herbal decoction OR herbal prescription OR Pharmacoacupuncture OR Pharmaco-acupuncture OR Pharmacopuncture	22,600
#3	Epidemiologic Studies OR Retrospective cohort OR Cohort studies OR Cohort Study OR Studies, Cohort OR Study, Cohort OR Cohort Studies OR Case-Control studies OR Case-Control Study OR Cross-sectional studies OR Cross-Sectional Study OR Insurance Claim Review OR nationwide study OR nationwide cohort OR Sample cohort OR claim data OR national health insurance OR national health information OR population based OR matched cohort OR Propensity Score OR health insurance review OR claim database OR claims database OR real-world OR real world OR Hospital information system	193,126
#4	#1 AND #2 AND #3	6

	**AMED (20220525) searches**	**Results**
#1	heart failure OR cardiac failure OR myocardial failure OR heart insufficiency OR cardiac insufficiency OR Cardiomyophathies OR Heart Decompensation OR Decompensation, Heart OR Congestive Heart Failure OR Ventricular Dysfunction OR Heart Failure, Congestive	1,034
#2	Medicine, Chinese Traditional OR Medicine, Kampo OR Medicine, Korean Traditional OR traditional Korean medicine OR traditional Chinese medicine OR traditional oriental medicine OR Kampo medicine OR Chinese patent medicine OR Acupuncture OR Acupuncture Therapy OR Auriculotherapy OR Acupuncture, Ear OR Acupressure OR Electroacupuncture OR Acupuncture Points OR dry needling OR acupoint OR trigger point OR dry needling OR ear acupuncture OR electro-acupuncture OR scalp acupuncture OR Head acupuncture OR acupotomy OR fire needling OR thread-embedding therapy OR Moxibustion OR moxa OR warm needling OR Cupping Therapy OR Complementary Therapies OR complementary medicine OR alternative	31,121

	medicine OR herbal medicine OR herbal decoction OR herbal prescription OR Pharmacoacupuncture OR Pharmaco-acupuncture OR Pharmacopuncture	
#3	Epidemiologic Studies OR Retrospective cohort OR Cohort studies OR Cohort Study OR Studies, Cohort OR Study, Cohort OR Cohort Studies OR Case-Control studies OR Case-Control Study OR Cross-sectional studies OR Cross-Sectional Study OR Insurance Claim Review OR nationwide study OR nationwide cohort OR Sample cohort OR claim data OR national health insurance OR national health information OR population based OR matched cohort OR Propensity Score OR health insurance review OR claim database OR claims database OR real-world OR real world OR Hospital information system	10,868
#4	#1 and #2 and #3	1

Supplementary Table S2. Frequency of used botanical drugs (herbal medicines) in the studies

Latin name	Chinese	Pinyin	Scientific name	Frequency of use (times)
Ginseng Radix	人蔘	Renshen	*Panax ginseng* C. A. Meyer	**8**
Astragali Radix	黃芪	Huangqi	*Astragalus membranaceus* Bunge	**7**
Salviae miltiorrhizae Radix et Rhizoma	丹蔘	Danshen	*Salvia miltiorrhiza* Bunge	
Glycyrrhiza radix et Rhizoma	甘草	Gancao	*Giycyrrhiza uralensis* Fisch.	**6**
Paeoniae Rubra Radix	赤芍藥	Chishao	*Paeonia lactiflora* Pall.	**5**
Liriopis seu Ophiopogonis Tuber	小葉麥門冬	Maidong	*Ophiopogon japonicus* Ker-Gawler	
Notoginseng Radix et Rhizoma	三七	Sanqi	*Panax notoginseng* (Burk.) F. H. Chen	
	蔘芪	TianSanqi	*Radix Pseudoginseng*	
Carthami Flos	紅花	Honghua	*Carthamus tinctorius* L.	
Chuanxiong Rhizoma	川芎	Chuanxiong	*Ligusticum chuanxiong* Hort.	
Persicae Semen	桃仁	Taoren	*Prunus persica* (L.) Batsch	**4**
Platycodonis Radix	桔梗	Jiegeng	*Platycodon grandiflorum* (Jacq.) A.DC.	
Angelicae Sinensis Radix	中國當歸	Danggui	*Angelica sinensis* (Oliv.) Diels	
Aconiti Lateralis Radix Praeparata	附子	Fuzi	*Aconitum carmichaelii* Debx.	
Poria Sclerotium	茯苓	Fuling	*Poria cocos* (Schw.) Wolf	
Paeoniae Radix Alba	白芍藥	Baishao	*Paeonia lactiflora* Pall.	
Zingiberis Rhizoma	乾薑	Ganjiang	*Zingiber officinale* Rosc.	
Cinnamomi Ramulus	桂枝	Guizhi	*Cinnamomum cassia* Presl	
Rehmanniae Radix	生地黃	Dihuang	*Rehmannia glutinosa* (Gaertner) Liboschitz ex Steudel	
Schisandrae Fructus	五味子	Wuweizi	*Schisandra chinensis* (Turcz.) Baill.	
Cyathulae Radix	川牛膝	Chuanniuxi	*Cyathula officinalis* Kuan	
Bupleuri Radix	柴胡	Chaihu	*Bupleurum chinense* DC.	
Cinnamomi Cortex	肉桂	Rougui	*Cinnamomum cassia* Presl	**3**
Alismatis Rhizoma	澤瀉	Zexie	*Alisma orientale* (Sam.) Juzep.	
Rehmanniae Radix Preparata	熟地黃	Shudihuang	*Rehmannia glutinosa* Liboschitz ex Steudel	
Scutellariae Radix	黃芩	Huangqin	*Scutellaria baicalensis* Gerogi	

Atractylodis Macrocephalae Rhizoma	白朮	Baizhu	*Atractylodes macrocephala* Koidz.	
Asini Corii Colla	阿膠	Ejiao	*Equus asinus* Linne	
Zizyphi Frucuts	大棗	Dazao	*Ziziphus jujuba* Mill.	
Zingiberis Rhizoma Recens	生薑	Shengjiang	*Zingiber officinale* Rosc.	
Ziziphi Spinosae Semen	酸棗仁	Suanzaoren	*Ziziphus jujuba* Mill. var. spinosa (Bge.) Hu ex H.F.Chou	
Allii Macrostemi Bulbus	薤白	Xiebai	*Allium macrostemon* Bge.	**2**
Bomeolum	氷片	Biangpian	*Dryobalanops aromatica* Gaertner	
Pinelliae Tuber	半夏	Banxia	*Pinellia ternata* Breitenbach	
Glycyrrhizae Preparata Radix	炙甘草	Zhigancao	*Glycyrrhiza uralensis* Fisch.	
Codonopsis Pilosulae Radix	黨參	Dangshen	*Codonopsis pilosula* (Franch.) Nannf.	
Trichosanthis Fructus	瓜蔞實	Gualou	*Trichosanthes kirilowii* Maxim.	
Dalbergiae Odoriferae Lignum	降香	Jiangxiang	*Dalbergia odorifera* T. Chen	
Ponciri Fructus Immaturus	枳實	Zhishi	*Poncirus trifoliata* Rafinesque	
Citri Reticulatae Pericarpium Viride	青皮	Qingpi	*Citrus reticulata* Blanco	
Cimicifugae Rhizoma	升麻	Shengma	*Cimicifuga heracleifolia* Kom.	
Ginseng Radix Rubra	紅蔘	Hongshen	*Panax ginseng* C. A. Meyer	
Agrimoniae Herba	仙鶴草	Xianhecao	*Agrimonia pilosa* Ledebour	
Gingko	白果	Baiguo	*Ginkgo biloba* Linné	
Curcumae Longae Rhizoma	薑黃	Jianghuang	*Curcuma longa* Linné	
Cannabis Semen	麻仁	Huomaren	*Cannabis sativa* Linné	
Anemarrhenae Rhizoma	知母	Zhimu	*Anemarrhena asphodeloides* Bunge	
Ligustici Rhizoma et Radix	藁本	Gaoben	*Ligusticum sinense* Oliv.	
Asparagi Radix	天門冬	Taindong	*Asparagus cochinchinensis* (Lour.) Merr.	
Scrophulariae Radix	玄參	Xuanshen	*Scrophularia buergeriana* Miquel	
Polygalae Radix	遠志	Yuanzhi	*Polygala tenuifolia* Willd.	
Platycladi Semen	柏子仁	Baiziren	*Platycladus orientalis* (L.) Franco	
Cinnabaris	朱砂	Zhusha	-	
Aurantii Frutus	枳殼	Zhiqiao	*Citrus aurantium* L.	
Corni Frutus	山茱萸	Shanzhuyu	*Cornus officinalis Siebold* et Zuccarini	
Dioscoreae Rhizoma	山藥	Shanyao	*Dioscorea opposita* Thunb.	
Moutan Radicis Cortex	牡丹皮	Mudanpi	*Paeonia suffruticosa* Andr.	
Atractylodis Rhizoma Alba	白朮	Baizhu	*Atractylodes japonica* Koidz.	**1**
Ephedra Herba	麻黃	Mahuang	*Ephedra sinica* Stapf	
Asari Radix et Rhizoma	細辛	Xixin	*Asarum sieboldii* Miq. var. seoulense Nakai	

Epimedii Herba	淫羊藿	Yinyanghuo	*Epimedium brevicornum* Maxim
Lepidii seu Descurainiae Semen	葶藶子	Tinglizi	*Lepidium apetalum* Willdenow
Atractylodis Rhizoma	蒼朮	Changzhu	*Atractylodes chinensis* (DC.) Koidz.
Leonuri Herba	益母草	Yimucao	*Leonurus japonicus* Houttuyn
Hedyotidis Herba	白花蛇舌草	Baihuasheshecao	*Hedyotis diffusa* Willdenow
Taraxaci Herba	蒲公英	Pugongying	*Taraxacum mongolicum* Handel-Mazzetti
Hordei Fructus Germinatus	麥芽	Maiya	*Hordeum vulgare* L.
Plantaginis semen	車前子	Chenqianzi	*Plantago asiatica* Linné
Moschus	麝香	Shexiang	*Moschus berezovskii* Flerove
Pheretima	地龍	Dilong	*Pheretima aspergillum* (E.Perrier)
Eupolyphaga	土鱉蟲	Tubiechong	*Eupolyphaga sinensis* Walker
Scolopendra	蜈蚣	Wugong	*Scolopendra subspinipes mutilans* L. Koch
Scorpio	全蝎	Quanxie	*Buthus martensii* Karsch
Pinelliae Rhizoma Fermentata	半夏麴	Banxia	*Pinellia ternata* Breitenbach
Poria Sclertum Cum Pini Radix	茯神	Fushen	*Poria cocos* Wolf
Uncariae Ramulus Cum Uncis	钩藤	Gouteng	*Uncaria sinensis* Havil.
Rhei Radix et Rhizoma	大黃	Dahuang	*Rheum palmatum* L.
Puerariae Radix	葛根	Gegen	*Pueraria lobata* Ohwi
Eucommiae Cortex	杜仲	Duzhong	*Eucommia ulmoides* Oliv.
PrunellaeSpica	夏枯草	Xiakucao	*Prunella vulgaris* L.
Crataegi Fructus	山楂	Shanzha	*Crataegus pinnatifida* Bge.

Supplementary Table S3. Analysis of botanical drug (herbal medicine) regimens

Study ID	Intervention (Formulation)	Manufacturing company	Capacity	Dosage/Frequency (per day)	Treatment period
Tai 2022 [23]	Any type of herbal prescription, including Fuzi	NA	NA	Classified into 5 groups according to the dosage of Fuzi (15/36/100/150/200 g)	Classified into 5 groups according to the time interval of Fuzi (0·5/1/1·5/2/2·5/3 years)
Guan 2022 [24]	Shenmai injection	NA	NA	NA	more than 7 days
Komagamine 2021 [25]	Kampo medications that can cause or exacerbate heart failure (Licorice composition, Ginseng composition, Ephedra composition, Aconite composition)	NA (Information about prescribed drugs, including Kampo medications, was obtained from a comprehensive medication list compiled by pharmacists during the course of routine care.)	NA	NA	NA
Yu 2019 [27]	Danshen dripping pills, Danshen polyphenolate injection, etc.<br>Xueshuantong injection, Sanqi Tonghu capsule, etc.<br>Qiliqiangxin capsule, Qishenyiqi Dripping pills, etc.<br>Ginkgo leaf capsule; Ginkgo leaf extract and dipyridamole injection, etc.<br>Safflower yellow injection; Safflower extraction, etc.	NA	NA	NA	Classified into 4 group (0/1/2-9/>9)
Huang 2019 [31]	NA	NA	NA	NA	NA
Sui 2018 [28]	SQLXF (Ginseng, 20 g; Astragalus, 20 g; Cassia twig, 10 g; Epimedium, 15 g; Semen lepidii, 20 g; Atractylodes, 15 g; Motherwort, 15 g; Poria, 20 g; Atractylodes, 15 g; Motherwort, 15 g; Salvia, 15 g; Agrimony, 30 g; licorice, 10 g)	Donghua Decoction Machine: YF12/3+1, Beijing, China	150 ml	300 ml/day	6 months
Guohua 2018 [33]	Shengmai, Dahong, Ligustrazine injection (main treatment) + HM	NA	NA	NA	(Admission stage) TCM injection (main treatment: high Ex group >14 d; low Ex group 7–13 d) + WM vs. WM (Follow-up stage) HM (patent medicine or decoction: low Ex 28 d to 3 m; medium Ex 3–6 m; high Ex >6 m) + WM vs. WM
Wulin 2018 [34]	Shengmai, Danhong, Ligustra injection + HM	NA	NA	NA	Low exposure group: 28 d and <3 months<br>Medium exposure group: 3 months and <6 months<br>High exposure group: ≥6 months

Tsai 2017 [29]	Zhi-Gan-Cao-Tang, Sheng-Mai-San, Zhen-Wu-Tang, Suan-Zao-Ren-Tang, Tian-Wang-Bu-Xin-Dan, Xue-Fu-Zhu-Yu-Tang, Ji-Sheng-Shen-Qi-Wan, Bu-Zhong-Yi-Qi-Tang, Si-Ni-Tang, Liu-Wei-Di-Huang-Wan	NA	NA	NA	NA
Tsai 2017 [30]	Zhi-Gan-Cao-Tang, Sheng-Mai-San, Zhen-Wu-Tang, Suan-Zao-Ren-Tang, Bu-Zhong-Yi-Qi-Tang, Si-Ni-Tang, Tian-Wang-Bu-Xin Dan, Xue-Fu-Zhu-Yu-Tang, Liu-Wei-Di-Huang-Wan, Yang-Xin-Tang	NA	NA	NA	NA
Liu 2022 [32]	Tian-Ma-Gou-Teng-Yin, Xue-Fu-Zhu-yu-Tang, Gou-Teng-San, Jia-Wei-Xiao-Yao-San, Zhi-Gan-Cao-Tang, Ji-Sheng-Shen-Qi-Wan,Liu-Wei-Di-Huang-Wan, Zhi-Bai-Di-Huang-Wan, Bu-Yang-Huan-Wu-Tang, Qi-Ju-Di-Huang-Wan	NA	NA	(Multi) 5·35–8·70 g/day (Single) 0·64–2·96 g/day	(Multi) 10·12–12·22 days (Single) 10·48–11·89 days

NA, not applicable; SQLXF, Shen Qi Li Xin Formula.
